# Vitamin D status & associations with inflammation in older adults

**DOI:** 10.1371/journal.pone.0287169

**Published:** 2023-06-28

**Authors:** Eamon Laird, Aisling M. O’Halloran, Anne M. Molloy, Martin Healy, Nollaig Bourke, Rose Anne Kenny

**Affiliations:** 1 School of Physical Education and Sports Science, University of Limerick, Limerick, Ireland; 2 School of Medicine, Trinity College Dublin, Dublin, Ireland; 3 The TILDA Study, School of Medicine, Trinity College Dublin, Dublin, Ireland; Shahid Beheshti University of Medical Sciences, ISLAMIC REPUBLIC OF IRAN

## Abstract

Research studies have observed associations of vitamin D with inflammation but data in representative older adult studies is lacking. We aimed to investigate the association of C-reactive protein (CRP) with vitamin D status in a representative sample of the older Irish population. The concentrations of 25-hydroxyvitamin D (25(OH)D) and CRP was measured in 5,381 community dwelling Irish adults aged ≥50 years from the Irish Longitudinal Study on Ageing (TILDA). Demographic, health and lifestyle variables were assessed by questionnaire and categorical proportions of CRP were generated by vitamin D status and age. Multi-nominal logistic regression was used to investigate the association of 25(OH)D and CRP status. The prevalence (mean; 95% confidence interval (95% CI)) of normal CRP status (0–5 mg/dL) was 83.9% (82.6–85.0%), elevated status (5–10 mg/dL) 11.0% (9.9–12.0%) and high status (>10 mg/dL) was 5.1% (4.5–5.8%). Mean (95% CI) CRP concentrations were lower in those with normal vs. deficient 25(OH)D status (2.02 mg/dL (1.95–2.08) vs. 2.60 mg/dL (2.41–2.82); p<0.0001). In a logistic regression analysis, those with insufficient or sufficient 25(OH)D status were less likely to have a high CRP status compared to those with deficient 25(OH)D status (insufficient: coefficient (CE) -0.732, 95% CI -1.12–0.33, p<0.0001; sufficient: CE -0.599, 95% CI -0.95–0.24, p = 0.001). In conclusion older adults with deficient vitamin D status had higher levels of inflammation as measured by CRP. Given that inflammation is an important pathological driver of chronic diseases of ageing, and that emerging evidence suggests that vitamin D therapy can reduce inflammation in some disease settings, optimising vitamin D status could represent an effective low risk/low-cost pathway to modulate inflammation in community dwelling older adults.

## Introduction

Vitamin D is a seco-steroid hormone that is essential for bone and musculoskeletal health [1] and has also been associated with chronic conditions such as cardiovascular disease (CVD), diabetes and cancer in observational and prospective studies [2, 3]. Purported mechanisms for these associations include the modulation of inflammation and preservation of endothelial function via effects of vitamin D on asymmetric dimethylarginine kinetics [4]. The main source of vitamin D is skin synthesis following exposure to ultraviolet B (UVB) light, however at far latitude countries, this is limited to the summer months with a sole reliance on dietary intakes and supplementation during the winter period [1, 5]. Importantly, few foods actually contain vitamin D (oily fish, mushrooms, etc.) in sufficient concentrations and these are infrequently consumed [5], whilst Ireland does not have a mandatory vitamin D food fortification policy resulting in poor dietary intakes of the vitamin [6]. Current estimates of vitamin D deficiency within middle aged and older Irish adults is 1 in 8 which increases to nearly 1 in 2 for those aged >85 yrs [7, 8]. These high rates of deficiency are of particular concern given the associations of vitamin D with immune function.

Vitamin D has been demonstrated to modulate the immune system via regulation of cell signalling pathways through the vitamin D receptor (VDR) which is present on a number of immune cells including monocytes, T lymphocytes and macrophages [9, 10]. Vitamin D has also been observed to regulate the proliferation of these cells and to influence the production of inflammatory cytokines such as Interleukin-6 (IL-6) and tumour necrosis factor alpha (TNF-alpha) [11]. Regulation of inflammation and cytokine expression is of crucial importance given the hypothesis of ‘inflamm-aging’–with increased age the shift toward a more pro-inflammatory state can lead to chronic low level grade inflammation and a slow accumulation of damage, with subsequent progression to chronic disease [12]. Vitamin D may also play a significant role related to immune function in the context of respiratory infection. Recent cross-sectional and randomized controlled trials (RCTs) have shown that low vitamin D status has been associated with a higher risk of infection, and vitamin D supplementation has been associated with reduced symptoms and antibiotic use [13–15]. These associations are particularly pertinent given the context of the COVID-19 pandemic where vitamin D has been associated with COVID mortality and severity of the immune response in older adults in some studies [16–18] though there is insufficient evidence, and it needs more clarification.

C-reactive protein (CRP) is an acute phase inflammatory protein produced by the liver in response to inflammation and mildly raised levels have been observed to be a significant predictor of CVD and mortality [19–22]. Thus, agents that could reduce or influence CRP production may also have a positive role in CVD and chronic disease prevention. In a meta-analysis of RCTs of vitamin D and CRP it was observed that vitamin D supplementation significantly decreased circulating high sensitivity CRP concentrations with a more pronounced effect in those with CRP concentrations >5 mg/L [23]. However, a more recent meta-analysis reported that vitamin D supplementation had no effect on CRP, Interleukin-10 or other cytokines [24]. Though the studies included were small and mostly in clinical populations. Importantly, few data exist examining the associations of vitamin D with CRP at a population level as these studies have mainly been performed in sub-groups [4, 25, 26]. Even fewer data exists in representative older populations [27] and there are currently no reports related to examining any casual associations the older Irish population. Thus, in this paper we aim to examine the association of vitamin D status with CRP status (measure of inflammation) in a large population representative study of older Irish adults.

## Materials and methods

### Study population

The study was approved by the Faculty of Health Sciences Research Ethics Committee at Trinity College Dublin, and all participants gave informed written consent. All experimental procedures adhered to the Declaration of Helsinki and all assessments were performed by trained research nurses. Anonymized data and materials have been made publicly available at the Irish Social Science Data Archive based in University College Dublin and the Interuniversity Consortium for Political and Social Research based in the University of Michigan and can be accessed at www.tilda.ie. Participants were part of the Irish Longitudinal Study on Ageing (TILDA), a nationally population representative cohort of community-dwelling adults aged ≥50 years. As described previously [7, 28], the first wave of data collection was conducted between October 2009 and July 2011 and all household residents aged ≥50 years were eligible to participate and these were stratified by socioeconomic group and geography to maintain a population representative sample (latitude 50–55°N). Clusters were selected with a probability proportional to the number of individuals aged ≥50 years in each cluster.

### Blood samples

Frozen non-fasting total plasma samples were accessed for the blood biomarker measurements. The concentration of total 25(OH)D (including D2 & D3) were quantified by LC-MS/MS with a validated method (Chromsystems Instruments and Chemicals GmbH; MassChrom 25-OH-Vitamin D3/D2) in the Biochemistry Department of St James’s Hospital (accredited to ISO 15189). The quality and accuracy of the method was monitored by the use of internal quality controls, participation in the Vitamin D External Quality Assessment Scheme (DEQAS) and the use of the National Institute of Standards and Technology (NIST) 972 vitamin D standard reference material. The respective inter- and intra-assay coefficients of variation (CV) were 5.7% and 4.5%. 25(OH)D is the recognised biomarker of vitamin D and is used in the assessment of the circulatory blood status. Vitamin D deficiency, insufficiency, and sufficiency were defined as <30, 30 to 50 and >50 nmol/L, respectively [29]. CRP concentrations were measured on a Roche Cobas c 701 analyser with a proprietary immunoturbidimetric assay (Roche Diagnostics Ireland, Tina-quant® C-Reactive Protein 3rd Gen). The respective inter- and intra-assay CV were 6.3% and 7.0%. Normal, elevated and high CRP were defined as 0–5, 5–10 and >10 mg/dL. Creatinine was measured by an enzymatic method traceable to isotope-dilution mass spectrometry (Roche Creatinine plus ver.2, Roche Diagnostics, Basel Switzerland) and the inter-assay CV was <5%. Glycated haemoglobin (HbA1c) concentration was measured by reversed-phase cation exchange chromatography using an ADAMS A1c HA-8180V analyser which is traceable to the internationally agreed standard developed by the International Federation of Clinical Chemistry (IFCC).

### Demographics

Demographic data was collected via a computer-aided personal interview and health assessment. Information included age, sex, education (categorized to primary, secondary or tertiary/higher), currently smoking (yes/no), CAGE alcohol score (yes/no problematic consumption) and self-reported presence of chronic disease including diabetes, stroke, heart failure, heart attack, hypertension, angina, heart murmur, transient ischemic attack and irregular heart rhythm (grouped into a categorical variable of 0,1,2 or 3/more chronic conditions). Obesity was measured as a body mass index (BMI) >30 kg/m^2^ while physical activity levels were classified using the International Physical Activity Questionnaire (IPAQ) categories: physically active (minimally or health enhancing physically active) versus physically inactive (inactive or insufficiently active).

### Statistical analyses

Weighted geometric means are presented along with concentrations of CRP categorized by normal, elevated and high levels and by population demographics. The weights used in the analysis were derived specifically for those who provided a blood sample; these were calculated by multiplying the base interview weight for a given participant by the inverse of the probability that the participant provided a blood sample (probability calculated using a logistic regression model). Pairwise comparisons by CRP category were computed across variables of interest and a Bonferroni correction for multiple comparisons was applied. The prevalence of CRP status by vitamin D concentrations and age groups within the population were computed and comparison of CRP concentrations by vitamin D status was examined by ANOVA with Bonferroni correction. Multi-nominal logistic regression modelled the predictors of elevated and high CRP (using normal as the reference category) with variables including vitamin D status, age category, sex, educational attainment, obesity, smoking, alcohol issue, physical activity, number of chronic diseases, creatinine and HBA1c concentrations. All analyses were carried out using STATA 14 (StataCorp, College Station, TX).

## Results

At baseline, 8,175 adults completed a computer-aided personal interview (CAPI), representing a response rate of 62%. Approximately 72.1% (n = 5,895) consented to, and participated in, a health assessment and 5,381 participants provided a blood sample for both a 25-hydroxyvitamin D (25(OH)D) and CRP measurement. Of those included in the study, the mean age (95% Confidence Intervals (CI)) was 62.9 years (50–98) and 53.5% were female. The mean BMI was 28.6 kg/m^2^, the rate of obesity was 33.9%, while 70.8% were physically active. Further information on the demographic details of the TILDA participants and the full breakdown regarding the vitamin D status of the population have been detailed extensively elsewhere (7). In short, 13% of the total population was deficient, with higher levels of deficiency in the oldest old, lower education, poorer socio-economic status and smokers while the distribution of supplement users was 8.5% (7). The estimated population prevalence of CRP status is presented in **Table 1**. The mean (95% CI) CRP of the total population was 3.30 mg/dL (3.05–3.55) and the prevalence (95% CI) of normal status was 83.9% (82.6–85.0%), elevated status 11.0% (9.99–12.0%) and high status was 5.1% (4.5–5.8%). A full age breakdown by sex is given in **S1, S2 Figs**. Overall, those who were younger, male, had tertiary education, were not obese, a non-smoker, and had less than three chronic diseases had significantly lower mean CRP concentrations. In terms of high CRP status (>10 mg/dL), again those aged ≥75 years compared to 50–64 year olds, primary education vs tertiary, obese vs non obese, physically inactive vs active and those with three or more chronic conditions vs less all had significantly higher proportions with high CRP (**Table 1**).

**Table 1:** Distribution of CRP concentrations and weighted prevalence of status by demographics in older Irish adults.

		CRP	Normal	Elevated	High
		Mean	0–5 mg/dL	>5–10 mg/dL	>10 mg/dL
	Subjects	(*n* = 5,381)	(*n* = 4,573)	(n = 554)	(*n* = 254)
Characteristic	*n*	mg/dL (95% CI)	% (95% CI)	% (95% CI)	% (95% CI)
**Age**					
50–64 y	3,263	2.03 (1.97–2.10)	85.5 (83.9–86.8)	10.6 (9.3–11.9)	3.9 (3.2–4.7)
65–74 y	1,425	2.23 (2.12–2.34)	83.5 (81.0–85.6)	10.8 (9.0–12.8)	5.7 (4.4–7.1)
≥75 y	693	2.54 (2.37–2.76)	79.1 (75.4–82.2)	12.4 (9.8–15.4)	8.5 (6.3–11.4)
**Sex**					
Male	2,502	2.08 (2.00–2.15)	85.3 (83.5–86.8)	10.0 (8.7–11.5)	4.7 (3.8–5.6)
Female	2,879	2.25 (2.17–2.33)	82.6 (80.9–84.0)	11.8 (10.5–13.2)	5.6 (4.7–6.7)
*p*		0.001	0.0146	0.057	0.162
**Education**					
Primary	1,372	2.52 (2.39–2.66)	77.6 (74.9–80.1)	15.0 (12.8–17.4)	7.4 (5.9–9.1)
Secondary	2,222	2.11 (2.03–2.19)	85.6 (83.9–87.1)	9.5 (8.2–10.8)	4.9 (4.0–5.9)
Tertiary	1,787	1.84 (1.77–1.92)	88.9 (87.1–90.4)	8.5 (7.1–10.1)	2.6 (1.8–3.5)
**Obesity**					
BMI >30 kg/m^2^	1,817	2.86 (2.74–2.99)	75.1 (72.8–77.2)	17.5 (15.6–19.6)	7.3 (6.1–8.7)
BMI <30 kg/m^2^	3,550	1.86 (1.81–1.92)	88.6 (87.2–89.8)	7.4 (6.4–8.5)	3.9 (3.1–4.8)
*p*		<0.0001	<0.0001	<0.0001	<0.0001
**Physical activity**					
Physically active	3,777	2.01 (1.95–2.07)	86.8 (85.5–88.0)	9.3 (8.2–10.3)	3.9 (3.2–4.6)
Physically inactive	1,558	2.54 (2.41–2.68)	77.4 (74.9–79.8)	14.6 (12.5–16.8)	8.0 (6.5–9.5)
*p*		<0.0001	<0.0001	<0.0001	<0.0001
**Smoking**					
Smoker	841	2.53 (2.37–2.70)	78.4 (74.9–81.4)	14.8 (12.1–18.0)	6.8 (5.0–8.9)
Non-smoker	4,540	2.09 (2.03–2.15)	85.2 (83.9–86.3)	10.0 (9.0–11.1)	4.8 (1.0–5.5)
*p*		<0.0001	<0.0001	0.0004	0.0385
**Problematic alcohol**					
No	4,208	2.17 (2.10–2.23)	84.1 (82.7–85.3)	10.4 (9.3–11.6)	5.5 (4.7–6.3)
Yes	631	2.00 (1.88–2.14)	85.4 (81.9–88.3)	10.9 (8.2–14.1)	3.7 (2.3–5.6)
*p*		0.034	0.441	0.8106	0.09
**Number of chronic diseases**					
None	1,193	1.90 (1.81–1.99)	88.2 (85.8–90.2)	9.2 (7.3–11.3)	2.6 (1.7–3.8)
One	1,521	2.12 (2.02–2.23)	83.7 (81.5–85.8)	10.8 (9.0–12.6)	5.5 (4.2–6.9)
Two	1,255	2.18 (2.07–2.30)	84.1 (81.7–86.3)	10.9 (8.9–13.0)	5.0 (3.8–6.5)
Three or more	1,412	2.45 (2.33–2.58)	80.0 (0.77–82.2)	12.8 (10.9–14.9)	7.2 (5.7–8.7)

Weighted means and prevalence estimates with 95% CI. P-values indicate significant pairwise comparisons of the difference in proportion of column criteria-across row variables. CRP = C-reactive protein. BMI = Body mass index; CI = Confidence interval. Physical activity levels were defined by IPAQ categories whilst problematic alcohol was defined by CAGE score.

### CRP and vitamin D

**Fig 1:**
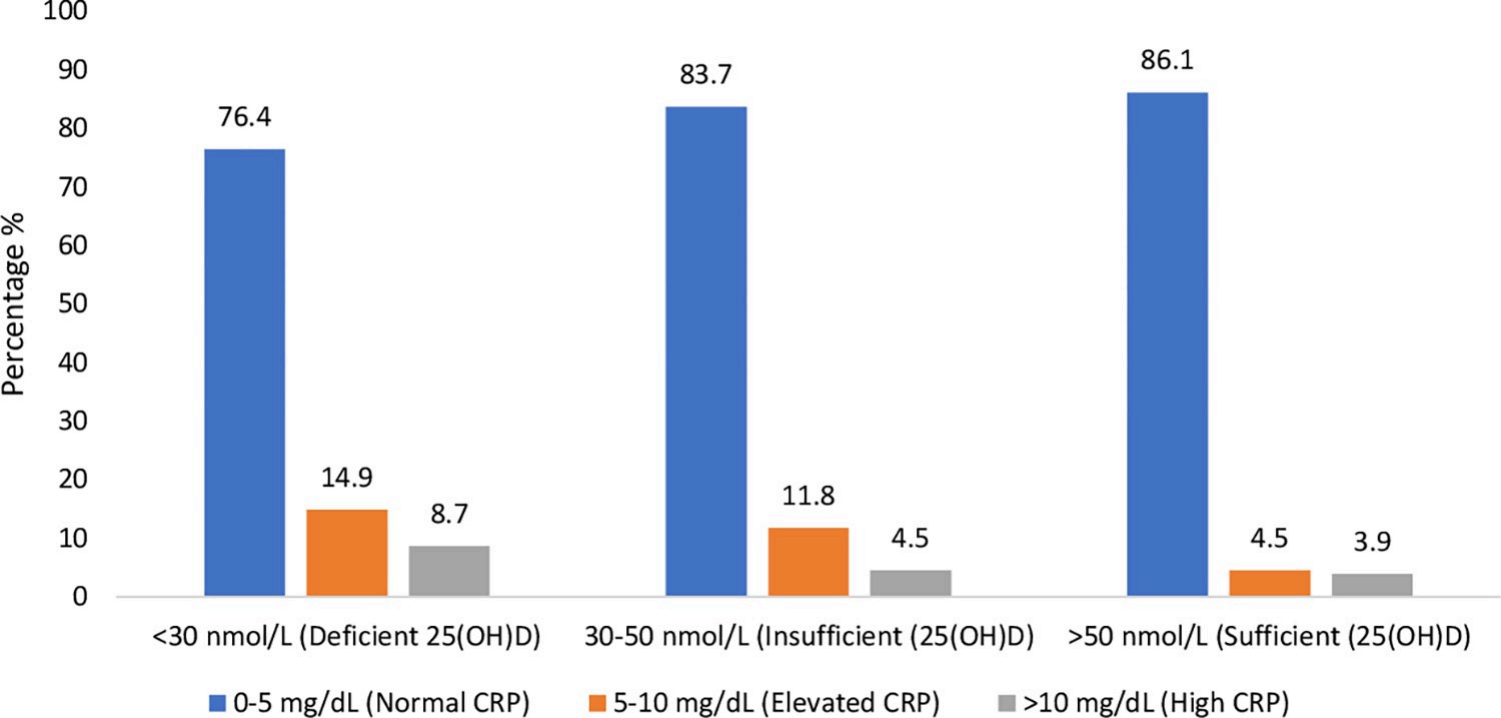
CRP profiles of older Irish adults by 25(OH)D status concentrations.

**Fig 2:**
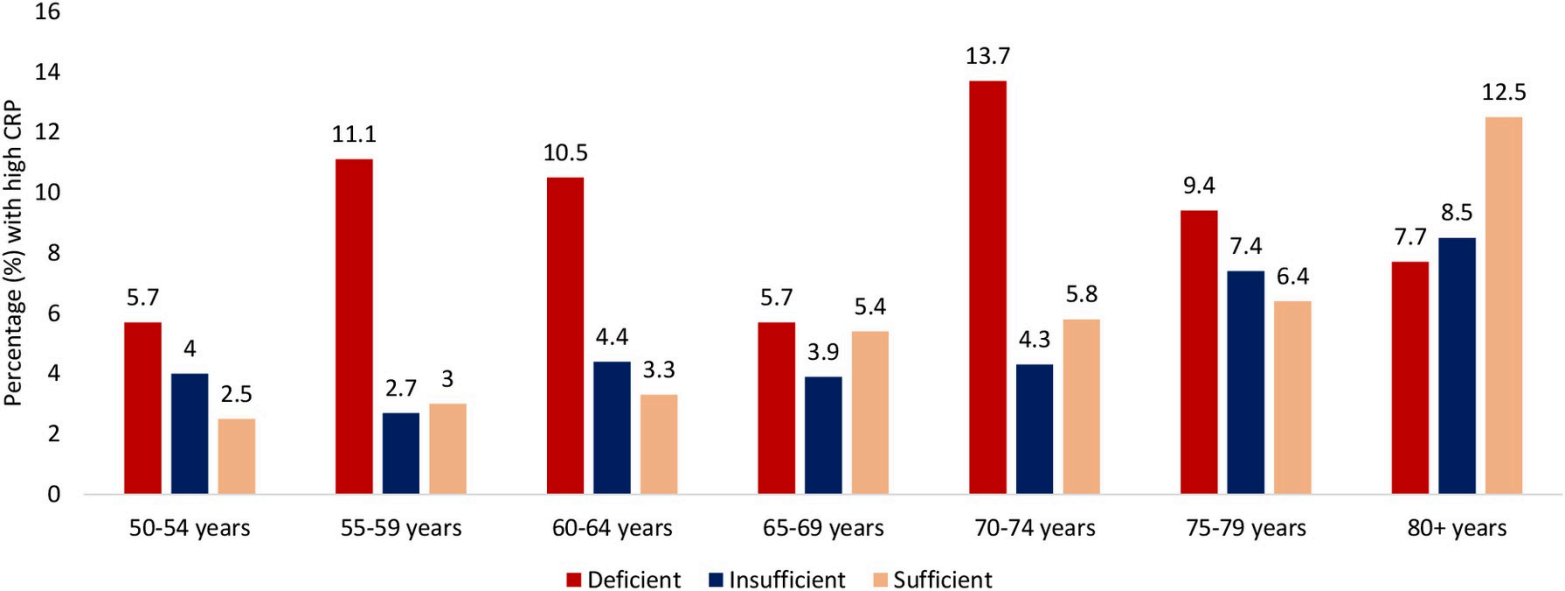
Percentage of the Irish older adult population with high CRP (>10 mg/dL) by age and vitamin D status.

In the total sample, mean (95% CI) CRP concentrations were significantly lower in those with normal vs. deficient 25(OH)D status (2.02 mg/dL (1.95–2.08) vs. 2.60 mg/dL (2.41–2.82); p<0.0001). The mean CRP concentration for insufficiency was 2.22 (2.13–2.32), significantly different from deficient and sufficient groups (P<0.001). There was also a shift in CRP status with increasing vitamin D category (**Fig 1**). As vitamin D status shifted from deficient to sufficient, the proportions of those with elevated or high CRP status decreased while the proportion with normal status increased. When examined by age (5-year profiles), similar results were observed, those with deficient 25(OH)D status had a higher proportion with high CRP status vs those with sufficient status until the age of ≥80 years (**Fig 2**). In a logistic regression analysis examining predictors of CRP status (**Table 2**), those with insufficient or sufficient 25(OH)D status were less likely to have a high CRP status compared to those with deficient 25(OH)D status (insufficient: coefficient (CE) -0.732, 95% CI -1.12,-0.33, p<0.0001; sufficient: CE -0.599, 95% CI -0.95,-0.24, p = 0.001). Other protective correlates included tertiary education. Correlate for increased risk of high CRP included obesity, smoking, female sex, physical inactivity, chronic conditions and high creatinine and HbA1c.

**Table 2:** The determinants of CRP concentrations (mg/dL) in older Irish adults.

Reference Category	Characteristic	Coefficient	Linearized *SE*	*p*	95% CI
**Normal CRP**	**Elevated CRP**			* *	
25(OH)D <30 nmol/L	25(OH)D 30–50 nmol/L	-0.189	0.152	0.214	(-0.48–0.10)
	25(OH)D >50 nmol/L	-0.243	0.142	0.088	(-0.52–0.03)
50–64 years	65–74 years	-0.951	0.125	0.448	(-0.34–0.15)
	≥75 years	0.109	0.160	0.498	(-0.20–0.42)
Male	Female	0.435	0.113	<0.0001	(0.21–0.65)
Primary education	Secondary education	-0.418	0.125	0.001	(-0.66–0.17)
	Tertiary education	-0.374	0.133	0.005	(-0.63–0.11)
Not obese	Obese	1.013	0.104	<0.0001	(0.80–1.21)
Non-smoker	Current smoker	0.586	0.129	<0.0001	(0.33–0.84)
No problematic alcohol	Problematic alcohol	0.124	0.152	0.416	(-0.17–0.42)
Physically active	Physically inactive	0.363	0.107	0.001	(0.15–0.57)
No chronic conditions	One chronic condition	0.055	0.152	0.716	(-0.24–0.35)
	Two chronic conditions	0.022	0.159	0.889	(-0.29–0.33)
	≥Three chronic conditions	0.010	0.161	0.946	(-0.30–0.32)
	Creatinine	0.004	0.002	0.023	(0.006–0.008)
	HbA1c	0.039	0.007	<0.0001	(0.02–0.05)
**Normal CRP**	**High CRP**				
25(OH)D <30 nmol/L	25(OH)D 30–50 nmol/L	-0.732	0.201	<0.0001	(-1.12–0.33)
	25(OH)D >50 nmol/L	-0.599	0.179	0.001	(-0.95–0.24)
50–64 years	65–74 years	0.151	0.172	0.38	(-0.18–0.48)
	≥75 years	0.399	0.206	0.053	(-0.004–0.80)
Female	Male	0.434	0.154	0.005	(0.13–0.73)
Primary education	Secondary education	-0.180	0.165	0.276	(-0.50–0.14)
	Tertiary education	-0.645	0.199	0.001	(-1.03–0.25)
Not obese	Obese	0.826	0.145	<0.0001	(0.54–1.11)
Non-smoker	Current smoker	0.561	0.180	0.002	(0.20–0.91)
No problematic alcohol	Problematic alcohol	-0.133	0.239	0.577	(-0.60–0.33)
Physically active	Physically inactive	0.393	0.147	0.008	(0.10–0.68)
No chronic conditions	One chronic condition	0.487	0.239	0.042	(0.17–0.95)
	Two chronic conditions	0.390	0.248	0.117	(-0.09–0.87)
	≥Three chronic conditions	0.440	0.246	0.074	(-0.04–0.92)
	Creatinine	0.007	0.002	0.002	(0.002–0.01)
	HbA1c	0.035	0.010	<0.0001	(0.01–0.05)

Multi-nominal logistic regression using normal CRP status as the reference category. 25(OH)D = 25-hydroxyvitamin D. CI = Confidence interval. Physical activity levels were defined by IPAQ categories whilst problematic alcohol was defined by CAGE score.

## Discussion

In this population study, sufficient vitamin D status was associated with a lower concentration of CRP even after adjustment for traditional risk factors. These findings (with previous RCT evidence) suggests that optimising vitamin D status to above deficient levels could help to modulate the inflammation pathway in community dwelling older adults. CRP is one of the major acute-phase proteins which can increase relatively rapidly in response to infection or injury and is considered to be a reliable and accurate measure of inflammatory response [30]. Few observational studies have examined vitamin D and inflammation in older adults. In the only other population representative study examining this issue, it was observed that low 25(OH)D concentrations were associated with high CRP in 5,870 older adults from the English Longitudinal Study of Ageing (ELSA) [27]. Similarly, in an observational study of 957 older Irish adults (>60 years) it was reported that low 25(OH)D concentrations (<25 nmol/L) vs. high (>75 nmol/L) was associated with a higher CRP and a skewed CRP:Interluekin-10 (IL-10) ratio indicating possible immune dysregulation [26]. However, some observational studies have found no associations such as the Framingham Offspring Study (n 1,381) though baseline 25(OH)D concentrations were higher, and 25(OH)D was measured using radioimmunoassay when compared to the gold standard technique used in TILDA study [31]. More recent randomized controlled trials (RCT) have demonstrated significant positive results. In a study of type 2 diabetics (n 50), two injections of 200,000 IU vitamin D resulted in a significant reduction in CRP concentrations [32]. In another RCT (n 90), ulcerative colitis patients received a 300,000 IU D3 injection which after 90 days resulted in a significant drop in CRP concentrations [33]. Furthermore a systematic review and meta-analysis of twenty trials reported that vitamin D intakes (through any route) were associated with lower CRP concentrations in type 2 diabetics [34] whilst another meta-analysis reported that vitamin D supplementation resulted in significantly lower CRP concentrations in patients with diabetic nephropathy [35]. As discussed earlier, other systematic reviews [23, 24] have reported mixed results as studies to date have been in mostly clinical populations, have used different dosage regimes, baseline blood levels have often been above deficiency levels and a range of different time-points have been used thus making it difficult to ascertain any true associations of vitamin D with CRP. In comparison, in the current study we have tried to examine this potential association in relatively normal free-living older adults, representative of the population at large and also taking into consideration health and other demographic factors to ascertain factors which may influence this relationship.

Despite these data there is debate as to whether changes in vitamin D status are because of its role in the acute phase response, or whether vitamin D is being lowered by the inflammation itself. Three studies have reported significant decreases in 25(OH)D blood concentration following an increase in biomarkers of inflammation [36–38]. However, in all three reports, patients had undergone major surgery, blood loss and fluid replacement. Interestingly, no direct mechanism has been proposed to explain why 25(OH)D concentrations may decrease with inflammation. Some have suggested that blood haemodilution, decreased synthesis of binding proteins and renal wasting of 25(OH)D that occurs in rapid acute illness or traumatic insults such as surgery [39] could be an explanation but these may not be a true representation of what occurs with low grade inflammation. Furthermore, other studies have reported no change in 25(OH)D concentrations during malarial infection or myocardial infarction, both of which would invariably lead to a significant increase in inflammation and thus a decrease in vitamin D status if the hypothesis was correct [40, 41].

CRP is the most commonly used laboratory marker of inflammation and can be produced in response to insult via pro-inflammatory cytokines [42]. In large clinical studies, CRP has been observed as a key predictor of CVD and all-cause mortality [20, 21, 43], depression [44] and more recently the need for mechanical ventilation in COVID-19 [45]. In terms of mechanisms of CRP and CVD risk, CRP may increase macrophage infiltration of adipose tissue and atherosclerotic lesions and influence plaque deposition [46]. CRP can also increase the concentration of prothrombin and D-dimer and increase the secretion of tissue factor by macrophages [47]. Elevated levels over long periods may also increase the risk of vascular dysfunction, renal fibrosis and tissue damage leading to increased risk of chronic disease [47]. The collective evidence from observational studies and trials indicate that CRP can both be a marker of long-term inflammation and be a potential contributor itself of chronic disease. Therefore, interventions such as optimizing blood vitamin D to sufficient status (>50 nmol/L) to try to lower CRP levels may have potential health benefits and could in part explain some of the observations of vitamin D with disease [48]. However, rates of vitamin D insufficiency (<50 nmol/L) are high both in older and younger adults [7, 49, 50] and in countries without a vitamin D food fortification policy, meeting a blood level target of 50 nmol/L is difficult to achieve. It has been estimated that to reach a blood 25(OH)D concentration of 50 nmol/L during the winter period, intakes of vitamin D from either food or supplements should be 25.0 μg [51]. Irish dietary vitamin D intakes have currently been estimated at 4.2 μg per day in adults aged 18–64 yrs old [52]. In contrast, in Finland which has a systematic policy of vitamin D food fortification of milk products and fat spreads, the proportions of vitamin D deficiency are <1% at the population level [53–55]. In recognition of the evidence regarding the poor dietary vitamin D intakes and inadequate vitamin D status of older Irish adults, the Food Safety Authority of Ireland has recently recommended all older Irish adults to consume a daily 15 μg/600 IU vitamin D supplement [6]. Future research will be able to investigate whether this will have any impact on the inflammatory status of the population.

Strengths of the study include a well characterised nationally representative older adult population, the use of LC-MS/MS gold standard as measurement of 25(OH)D status and adjustment for known established variables which can affect both 25(OH)D and CRP concentrations. Limitations include the cross-sectional design, single timepoint measure and CRP as the single measure of inflammation. It is also important to note that correlation is not causation and there maybe other confounding variables which may mediate the associations of 25(OH)D with CRP such as pollution or other environmental or social circumstances. Additionally, no information was available for dietary intakes or UVB exposure, however the focus of this paper was on the associations of 25(OH)D concentrations (regardless of the source) with CRP and thus the source of the vitamin D be it from supplements, diet or sun does not affect the outcome in this instance.

## Conclusion

In this study we observed that older adults with low-deficient vitamin D status had higher levels of inflammation as measured by CRP concentrations. Given that inflammation is an important pathological driver of chronic diseases of ageing, and that emerging evidence suggests that vitamin D therapy can reduce inflammation in some disease settings, optimising vitamin D status could represent an effective low risk/low-cost pathway to modulate inflammation in community dwelling older adults.

## Supporting information

S1 Fig Percentage of the Irish male older adult population with CRP profiles by age. (TIF)

S2 Fig Percentage of the Irish female older adult population with CRP profiles by age. (TIF)

## References

[1] Laird E, Ward M, McSorley E, Strain JJ, Wallace J. Vitamin D and bone health; Potential mechanisms. Nutrients. 2010; 2(7):693–724. doi: 10.3390/nu2070693 . 22254049 PMC3257679

[2] Pludowski P, Holick MF, Pilz S, Wagner CL, Hollis BW, Grant WB, et al. Vitamin D effects on musculoskeletal health, immunity, autoimmunity, cardiovascular disease, cancer, fertility, pregnancy, dementia and mortality-a review of recent evidence. Autoimmun Rev. 2013; 12(10):976–989. doi: 10.1016/j.autrev.2013.02.004 . 23542507

[3] Bouillon R, Marcocci C, Carmeliet G, Bikle D, White JH, Dawson-Hughes B, et al. Skeletal and extraskeletal actions of vitamin D: current evidence and outstanding questions. Endocr Rev. 2019; 40(4):1109–1151. doi: 10.1210/er.2018-00126 . 30321335 PMC6626501

[4] Ngo DT, Sverdlov AL, McNeil JJ, Horowitz JD. Does vitamin D modulate asymmetric dimethylarginine and C-reactive protein concentrations? Am J Med. 2010; 123(4):335–341. doi: 10.1016/j.amjmed.2009.09.024 . 20362753

[5] Scientific Advisory Committee on Nutrition. Vitamin D and health. 2016.

[6] Vitamin D Scientific Recommendations for Food-Based Dietary Guidelines For Older Adults In Ireland 2020. Food Safety Authority of Ireland accessed 17/11/22 https://www.fsai.ie/news#:~:text=A%20daily%2015%20%C2%B5g%20vitamin,this%20vitamin%20from%20sunlight%20exposure.

[7] Laird E, O’Halloran AM, Carey D, Healy M, O’Connor D, Moore P, et al. The prevalence of vitamin D deficiency and the determinants of 25 (OH) D concentration in older Irish adults: Data from The Irish Longitudinal Study on Ageing (TILDA). J Gerontol A Biol Sci Med Sci. 2018; 73(4):519–525. doi: 10.1093/gerona/glx168 . 28958047

[8] Laird E, Kenny RA. Vitamin D deficiency in Ireland: Implications for COVID-19. Results from the Irish Longitudinal Study on Ageing. April 4 2020. 10.38018/TildaRe.2020-05

[9] Di Rosa M, Malaguarnera M, Nicoletti F, Malaguarnera L. Vitamin D3: a helpful immuno-modulator. Immunology. 2011; 134(2):123–139. doi: 10.1111/j.1365-2567.2011.03482.x . 21896008 PMC3194221

[10] Veldman CM, Cantorna MT, DeLuca HF. Expression of 1,25-dihydroxyvitamin D3 receptor in the immune system. Arch Biochem Biophys. 2000; 374(2):334–338. doi: 10.1006/abbi.1999.1605 . 10666315

[11] Wu D, Lewis ED, Pae M, Meydani SN. Nutritional modulation of immune function: analysis of evidence, mechanisms, and clinical relevance. Front Immunol. 2019; 15(9):3160. doi: 10.3389/fimmu.2018.03160 . 30697214 PMC6340979

[12] Ferrucci L, Fabbri E. Inflammageing: chronic inflammation in ageing, cardiovascular disease, and frailty. Nat Rev Cardiol. 2018; 15(9):505–522. doi: 10.1038/s41569-018-0064-2 . 30065258 PMC6146930

[13] Ginde AA, Mansbach JM, Camargo CA. Association between serum 25-hydroxyvitamin D level and upper respiratory tract infection in the Third National Health and Nutrition Examination Survey. Arch Intern Med. 2009; 169(4):384–390. doi: 10.1001/archinternmed.2008.560 . 19237723 PMC3447082

[14] Jolliffe DA, Griffiths CJ, Martineau AR. Vitamin D in the prevention of acute respiratory infection: Systematic review of clinical studies. J Steroid Biochem Mol Biol. 2013; 136:321–329. doi: 10.1016/j.jsbmb.2012.11.017 . 23220552

[15] Bergman P, Norlin AC, Hansen S, Rekha RS, Agerberth B, Bjorkhem-Bergman L, et al. Vitamin D3 supplementation in patients with frequent respiratory tract infections: a randomised and double-blind intervention study. BMJ Open. 2012; 2(6):e001663. doi: 10.1136/bmjopen-2012-001663 . 23242238 PMC3533016

[16] Laird E, Rhodes J, Kenny RA. Vitamin D and inflammation: Potential implications for severity of COVID-19. Ir Med J. 2020; 113(5):1. . 32603576

[17] Gibbons JB, Norton EC, McCullough JS, Meltzer DO, Lavigne J, Fiedler VC, et al. Association between vitamin D supplementation and COVID-19 infection and mortality. Scientific Reports. 2022; 12(1):1–1. doi: 10.1038/s41598-022-24053-4 . 36371591 PMC9653496

[18] Rhodes JM, Subramanian S, Laird E, Kenny RA. Low population mortality from COVID‐19 in countries south of latitude 35 degrees north supports vitamin D as a factor determining severity. Aliment Pharmacol Ther. 2020; 51(12):1434–1437. doi: 10.1111/apt.15777 . 32311755 PMC7264531

[19] Dong Y, Wang X, Zhang L, Chen Z, Zheng C, Wang J, et al. High-sensitivity C reactive protein and risk of cardiovascular disease in China-CVD study. J Epidemiol Community Health. 2019; 73(2):188–192. doi: 10.1136/jech-2018-211433 . 30530521

[20] Buckley DI, Fu R, Freeman M, Rogers K, Helfand M. C-reactive protein as a risk factor for coronary heart disease: a systematic review and meta-analyses for the U.S. Preventive Services Task Force. Ann Intern Med. 2009; 151:483–495. doi: 10.7326/0003-4819-151-7-200910060-00009 . 19805771

[21] Halcox JP, Roy C, Tubach F, Banegas JR, Dallongeville J, Backer GD, et al. C-reactive protein levels in patients at cardiovascular risk: EURIKA study. BMC Cardiovasc Disord. 2014; 14:25. doi: 10.1186/1471-2261-14-25 . 24564178 PMC3943833

[22] Wilson PW, Pencina M, Jacques P, Selhub J, D’Agostino R, O’Donnell CJ. C-reactive protein and reclassification of cardiovascular risk in the Framingham Heart Study. Circ Cardiovasc Qual Outcomes. 2008; 1(2):92–97. doi: 10.1161/CIRCOUTCOMES.108.831198 . 20031795 PMC3033831

[23] Chen N, Wan Z, Han SF, Li BY, Zhang ZL, Qin LQ. Effect of vitamin D supplementation on the level of circulating high-sensitivity C-reactive protein: a meta-analysis of randomized controlled trials. Nutrients. 2014; 6:2206–2216. doi: 10.3390/nu6062206 . 24918698 PMC4073144

[24] Mazidi M, Rezaie P, Vatanparast H. Impact of vitamin D supplementation on C-reactive protein; a systematic review and meta-analysis of randomized controlled trials. BMC nutrition. 2018;4(1):1–1. doi: 10.1186/s40795-017-0207-6 . 32153865 PMC7050714

[25] Michos ED, Streeten EA, Ryan KA, Rampersaud E, Peyser PA, Bielak LF, et al. Serum 25-hydroxyvitamin D levels are not associated with subclinical vascular disease or C-reactive protein in the old order Amish. Calcif Tissue Int. 2009; 84(3):195–202. doi: 10.1007/s00223-008-9209-3 . 19148561 PMC2908302

[26] Laird E, McNulty H, Ward M, Hoey L, Wallace JM, McSorley E, et al. Vitamin D deficiency is associated with inflammation in older Irish adults. J Clin Endocrinol Metab. 2014; 99:1807–1815. doi: 10.1210/jc.2013-3507 . 24606079

[27] de Oliveira C, Biddulph JP, Hirani V, Schneider IJ. Vitamin D and inflammatory markers: cross-sectional analyses using data from the English Longitudinal Study of Ageing (ELSA). J Nutr Sci. 2017; 6e1. doi: 10.1017/jns.2016.37 . 28620476 PMC5465858

[28] Kearney PM, Cronin H, O’Regan C, Kamiya Y, Savva GM, Whelan B et al. Cohort profile: the Irish Longitudinal Study on Ageing. Int J Epidemiol. 2011; 40(4): 877–884. doi: 10.1093/ije/dyr116 . 21810894

[29] Scientific Committee to Review Dietary Reference Intakes for Vitamin D and Calcium, Institute of Medicine. Dietary Reference Intakes for Calcium and Vitamin D. Washington, DC: The National Academies Press; 2011.

[30] Sproston NR, Ashworth JJ. Role of C-reactive protein at sites of inflammation and infection. Front Immunol. 2018; 9:754. doi: 10.3389/fimmu.2018.00754 . 29706967 PMC5908901

[31] Shea MK, Booth SL, Massaro JM, Jacques PF, D’Agostino RB Sr, Dawson-Hughes B, et al. Vitamin K and vitamin D status: associations with inflammatory markers in the Framingham Offspring Study. American J of Epidemiol. 2008; 167(3):313–320. doi: 10.1093/aje/kwm306 . 18006902 PMC3151653

[32] Mirzavandi F, Talenezhad N, Razmpoosh E, Nadjarzadeh A, Mozaffari-Khosravi H. The effect of intramuscular megadose of vitamin D injections on E-selectin, CRP and biochemical parameters in vitamin D-deficient patients with type-2 diabetes mellitus: A randomized controlled trial. Complement Ther Med. 2020; 49:102346. doi: 10.1016/j.ctim.2020.102346 . 32147032

[33] Sharifi A, Hosseinzadeh-Attar MJ, Vahedi H, Nedjat S. A randomized controlled trial on the effect of vitamin D3 on inflammation and cathelicidin gene expression in ulcerative colitis patients. Saudi J Gastroenterol. 2016; 22(4):316–323. doi: 10.4103/1319-3767.187606 . 27488327 PMC4991203

[34] Mousa A, Naderpoor N, Teede H, Scragg R, de Courten B. Vitamin D supplementation for improvement of chronic low-grade inflammation in patients with type 2 diabetes: a systematic review and meta-analysis of randomized controlled trials. Nutrition Rev. 2018; 76(5):380–394. doi: 10.1093/nutrit/nux077 . 29490085

[35] Wang Y, Yang S, Zhou Q, Zhang H, Yi B. Effects of vitamin D supplementation on renal function, inflammation and glycemic control in patients with diabetic nephropathy: a systematic review and Meta-analysis. Kidney and Blood Press Res. 2019; 44(1):72–87. doi: 10.1159/000498838 . 30808855

[36] Louw JA, Werbeck A, Louw ME, Kotzw TJ, Cooper R, Labadarios D. Blood vitamin concentrations during the acute-phase response. Critical Care Med. 1992; 20(7):934–941. doi: 10.1097/00003246-199207000-00007 . 1617986

[37] Reid D, Toole BJ, Knox S, Talwar D, Harten J, O’Reilly DS, et al. The relation between acute changes in the systemic inflammatory response and plasma 25-hydroxyvitamin D concentrations after elective knee arthroplasty. Am J Clin Nutr. 2011; 93(5):1006–1111. doi: 10.3945/ajcn.110.008490 . 21411617

[38] Waldron JL, Ashby HL, Cornes MP, Bechervaise J, Razavi C, Thomas OL, et al. Vitamin D: a negative acute phase reactant. J Clin Pathol. 2013; 66(7):620–622. doi: 10.1136/jclinpath-2012-201301 . 23454726

[39] Quraishi SA, Camargo CA Jr. Vitamin D in acute stress and critical illness. Curr Opinion Clin Nutr Metab Care. 2012; 15(6):625–634. doi: 10.1097/MCO.0b013e328358fc2b . 23075939 PMC3751798

[40] Barth JH, Field HP, Mather AN, Plein S. Serum 25 hydroxy-vitamin D does not exhibit an acute phase reaction after acute myocardial infarction. Ann Clin Biochem. 2012; 49(4):399–401. doi: 10.1258/acb.2011.011195 . 22543926

[41] Newens K, Filteau S, Tomkins A. Plasma 25-hydroxyvitamin D does not vary over the course of a malarial infection. Trans R Soc Trop Med Hyg. 2006; 100(1):41–44. doi: 10.1016/j.trstmh.2005.06.022 . 16171835

[42] Du Clos TW, Mold C. C-reactive protein: an activator of innate immunity and a modulator of adaptive immunity. Immuniol Res. 2004; 30(3):261–277. doi: 10.1385/IR:30:3:261 . 15531769

[43] Kaptoge S, Di Angelantonio E, Lowe G, Pepys MB, Thompson SG, Collins R, et al. C-reactive protein concentration and risk of coronary heart disease, stroke, and mortality: an individual participant meta-analysis. Lancet 2010. 375(99709):132–140. doi: 10.1016/S0140-6736(09)61717-7 . 20031199 PMC3162187

[44] Valkanova V, Ebmeier KP, Allan CL. CRP, IL-6 and depression: a systematic review and meta-analysis of longitudinal studies. J Affect Disord. 2013; 150(3):736–744. doi: 10.1016/j.jad.2013.06.004 . 23870425

[45] Herold T, Jurinovic V, Arnreich C, Lipworth BJ, Hellmuth JC, von Bergwelt-Baildon M, et al. Elevated levels of IL-6 and CRP predict the need for mechanical ventilation in COVID-19. J Allergy Clin Immunol. 2020; 146(1):128–136. doi: 10.1016/j.jaci.2020.05.008 . 32425269 PMC7233239

[46] Yousuf O, Mohanty BD, Martin SS, Joshi PH, Blaha MJ, Nasir K, et al. High-sensitivity C-reactive protein and cardiovascular disease: a resolute belief or an elusive link? J Am Coll Cardiol. 2013; 62:397–408. doi: 10.1016/j.jacc.2013.05.016 . 23727085

[47] Luan YY, Yao YM. The clinical significance and potential role of C-reactive protein in chronic inflammatory and neurodegenerative diseases. Front Immunol. 2018; 9:1302. doi: 10.3389/fimmu.2018.01302 . 29951057 PMC6008573

[48] Muscogiuri G, Altieri B, Annweiler C, Balercia G, Pal HB, Boucher BJ, et al. Vitamin D and chronic diseases: the current state of the art. Arch Toxicol. 2017; 91(1):97–107. doi: 10.1007/s00204-016-1804-x . 27425218

[49] Laird E, Shannon T, Crowley VE, Healy M. The benefits of utilising geo-mapping for visualising the vitamin D status of Dublin city and the surrounding urban districts. Ir J Med Sci. 2017; 186(4):807–813. doi: 10.1007/s11845-016-1517-4 . 27770264

[50] Scully H, Laird E, Healy M, Walsh JB, Crowley V, McCarroll K. Geomapping Vitamin D Status in a Large City and Surrounding Population-Exploring the Impact of Location and Demographics. Nutrients. 2020; 12(9):2663. doi: 10.3390/nu12092663 . 32878330 PMC7551618

[51] Cashman KD, Hill TR, Lucey AJ, Taylor N, Seamans KM, Muldowney S, et al. Estimation of the dietary requirement for vitamin D in healthy adults. Am J Clin Nutr. 2008; 88(6):1535–1542. doi: 10.3945/ajcn.2008.26594 . 19064513

[52] Hill TR, O’Brien MM, Cashman KD, Flynn A, Kiely M. Vitamin D intakes in 18-64-y-old Irish adults. Eur J Clin Nutr. 2004; 58(11):1509–1517. doi: 10.1038/sj.ejcn.1602001 . 15138462

[53] Jääskeläinen T, Itkonen ST, Lundqvist A, Erkkola M, Koskela T, Lakkala K, et al. The positive impact of general vitamin D food fortification policy on vitamin D status in a representative adult Finnish population: evidence from an 11-y follow-up based on standardized 25-hydroxyvitamin D data. Am J Clin Nutr. 2017; 105(6):1512–1520. doi: 10.3945/ajcn.116.151415 . 28490516

[54] Itkonen ST, Erkkola M, Lamberg-Allardt CJ. Vitamin D fortification of fluid milk products and their contribution to vitamin D intake and vitamin D status in observational studies—a review. Nutrients. 2018;10(8):1054. doi: 10.3390/nu10081054 . 30096919 PMC6116165

[55] Ikonen H, Lumme J, Seppälä J, Pesonen P, Piltonen T, Järvelin MR, et al. The determinants and longitudinal changes in vitamin D status in middle-age: a Northern Finland Birth Cohort 1966 study. EJCN. 2021;60(8):4541–53. https://link.springer.com/article/10.1007/s00394-021-02606-z . 34137914 10.1007/s00394-021-02606-z PMC8572212

