# Supplementary figures and images for: Vitamin D status & associations with inflammation in older adults
Source: PLoS One. 2023 Jun 28;18(6):e0287169. doi: 10.1371/journal.pone.0287169 (PMC10306176; doi:10.1371/journal.pone.0287169)

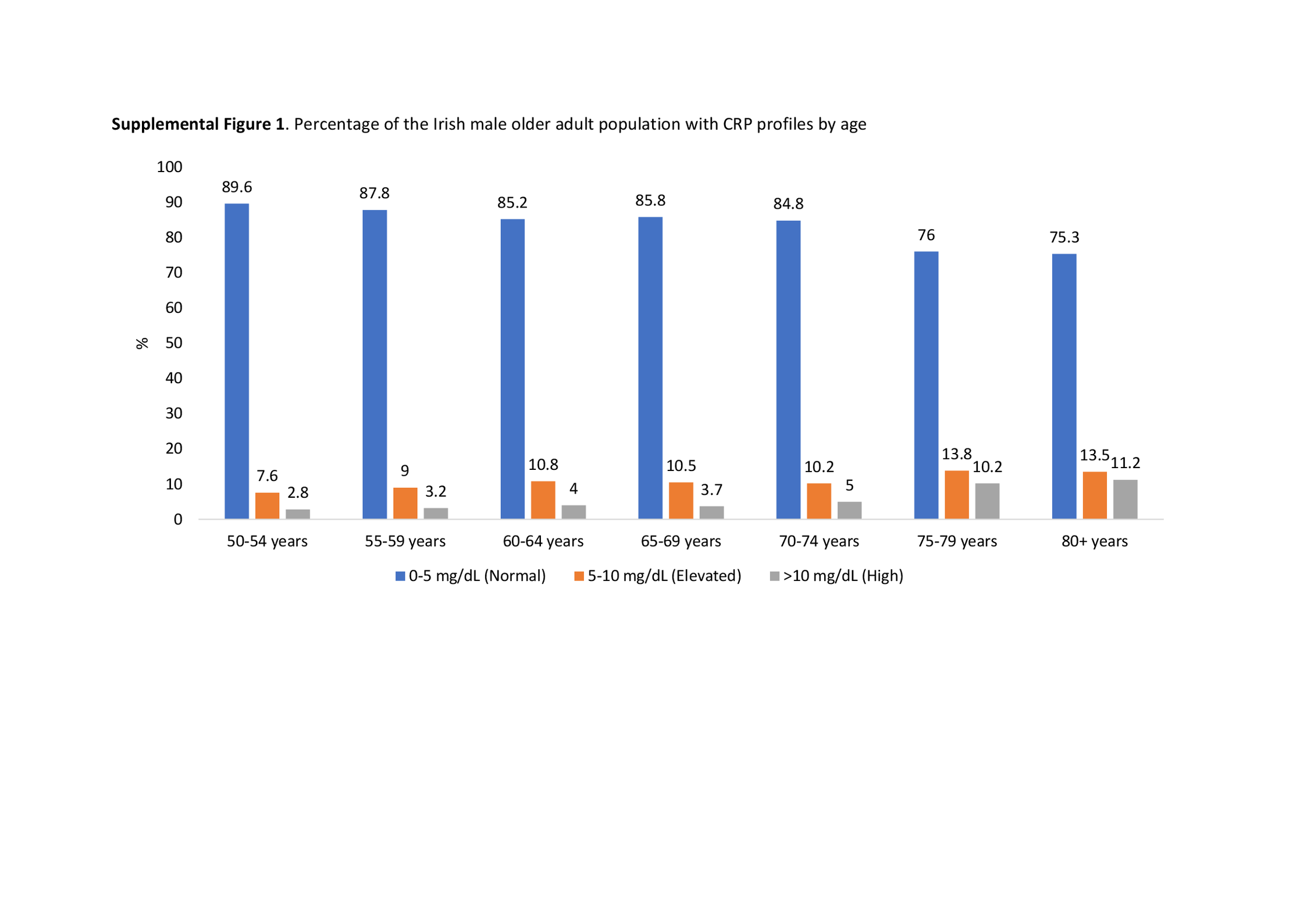

Supplement: S1 Fig — (TIF) [file pone.0287169.s001.tif]

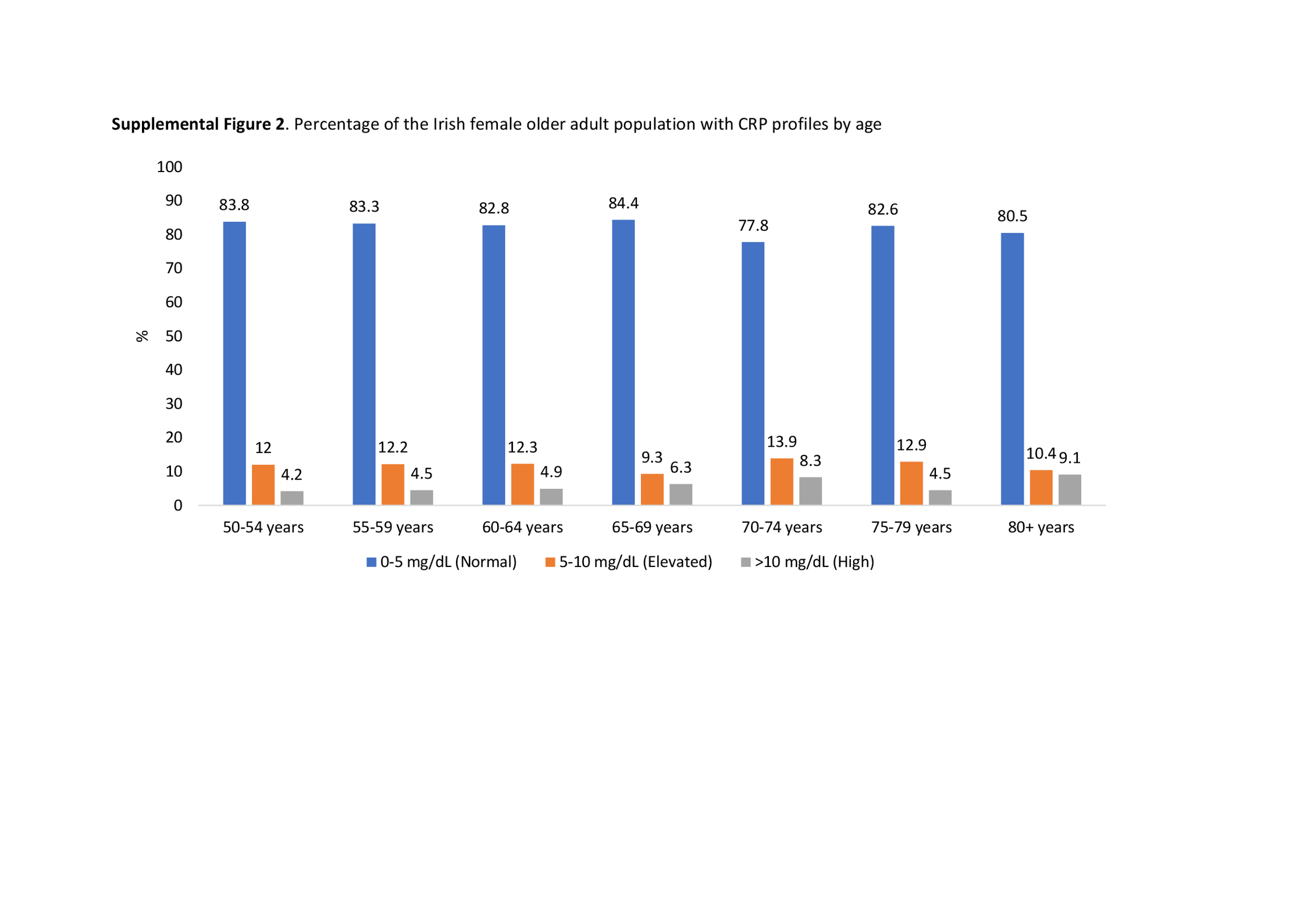

Supplement: S2 Fig — (TIF) [file pone.0287169.s002.tif]
